# Elevated *p*CO_2_ affects tissue biomass composition, but not calcification, in a reef coral under two light regimes

**DOI:** 10.1098/rsos.170683

**Published:** 2017-11-01

**Authors:** C. B. Wall, R. A. B. Mason, W. R. Ellis, R. Cunning, R. D. Gates

**Affiliations:** 1 Hawai‘i Institute of Marine Biology, School of Ocean and Earth Science and Technology, University of Hawai‘i at Mānoa, PO Box 1346, Kāne‘ohe, HI 96744, USA; 2 Northeastern University, 360 Huntington Avenue, Boston, MA 02115, USA

**Keywords:** ocean acidification, irradiance, scleractinian, biomass, energy reserves

## Abstract

Ocean acidification (OA) is predicted to reduce reef coral calcification rates and threaten the long-term growth of coral reefs under climate change. Reduced coral growth at elevated *p*CO_2_ may be buffered by sufficiently high irradiances; however, the interactive effects of OA and irradiance on other fundamental aspects of coral physiology, such as the composition and energetics of coral biomass, remain largely unexplored. This study tested the effects of two light treatments (7.5 versus 15.7 mol photons m^−2^ d^−1^) at ambient or elevated *p*CO_2_ (435 versus 957 µatm) on calcification, photopigment and symbiont densities, biomass reserves (lipids, carbohydrates, proteins), and biomass energy content (kJ) of the reef coral *Pocillopora acuta* from Kāne‘ohe Bay, Hawai‘i. While *p*CO_2_ and light had no effect on either area- or biomass-normalized calcification, tissue lipids gdw^−1^ and kJ gdw^−1^ were reduced 15% and 14% at high *p*CO_2_, and carbohydrate content increased 15% under high light. The combination of high light and high *p*CO_2_ reduced protein biomass (per unit area) by approximately 20%. Thus, under ecologically relevant irradiances, *P. acuta* in Kāne‘ohe Bay does not exhibit OA-driven reductions in calcification reported for other corals; however, reductions in tissue lipids, energy content and protein biomass suggest OA induced an energetic deficit and compensatory catabolism of tissue biomass. The null effects of OA on calcification at two irradiances support a growing body of work concluding some reef corals may be able to employ compensatory physiological mechanisms that maintain present-day levels of calcification under OA. However, negative effects of OA on *P. acuta* biomass composition and energy content may impact the long-term performance and scope for growth of this species in a high *p*CO_2_ world.

## 1. Introduction

Scleractinian corals are engineers of tropical coral reef ecosystems, directing the architecture and bioenergetics of these communities [1]. These ecosystems are, however, threatened by rapid seawater warming and ocean acidification (OA) associated with increasing concentrations of carbon dioxide (*p*CO_2_) in the atmosphere [2], which is predicted to double by the end of the century (650–850 µatm *p*CO_2_) [3]. Dissolution of atmospheric CO_2_ in the upper ocean alters the carbonate chemistry of seawater and reduces seawater pH and the saturation state of aragonite (Ω_arag_) [4]. These changes in seawater chemistry negatively impact many marine organisms, for example, by reducing rates of biogenic calcification in ecologically and economically important marine calcifiers [5,6]. The magnitude of OA effects on coral calcification, however, may be buffered by biological mechanisms (e.g. upregulation of internal pH) [7], environmental conditions (e.g. light, temperature, water motion) [8–11] and increasing energy available for metabolism (e.g. heterotrophy) [12,13].

Light availability impacts reef corals by modulating *Symbiodinium* spp. photosynthesis, which influences both the formation of skeleton [4] and the generation of lipid biomass [14] from translocated photosynthates [15,16]. Despite the importance of light to coral biology, the role of light in modulating coral responses to elevated *p*CO_2_ has only recently been considered [9]. Many OA experiments have been performed under low light levels (electronic supplementary material, table S1) that probably do not saturate photosynthesis and calcification rates, which may increase OA-sensitivity. Indeed, low light exacerbates the negative effects of high *p*CO_2_ on the growth of at least some corals [9,17], whereas increased light availability can mitigate negative effects of OA on growth observed at lower irradiances [18]. The role of light in modulating OA effects on skeletal growth is gaining attention; however, few studies have addressed whether other equally important aspects of coral physiology—such as tissue biomass growth and composition, and the allocation of energy resources—are impacted by *p*CO_2_ [19–21] and its interaction with light availability.

Understanding the interactive impacts of OA and light availability on coral tissue biomass is critically important, given that the quantity [22] and biochemical composition (e.g. lipids, carbohydrates, proteins) of biomass has important ecological implications for corals, including their response to environmental stress. In particular, lipids, which comprise approximately 30–45% of dry biomass [16], are a critical energy source in the early life history of reef corals [23], for parental provisioning of brooded larvae [24], and in adult corals recovering from bleaching [25]. Indeed, corals with greater lipid content [26] and/or tissue biomass [27] may avoid post-bleaching mortality.

The quantity and quality (e.g. lipid proportion or energy content) of tissue biomass may be impacted by OA as a response to altered metabolic demands or resource allocation. For instance, physiological stress from OA may increase the energetic costs of calcification and cellular homeostasis (e.g. ion transport, protein turnover) [28,29], in turn promoting the catabolism of lipid energy reserves to meet these demands [30]. Indeed, OA produces both positive and negative effects on coral biomass. Tissue biomass [31,32] (and lipid content [19]) can increase in some corals under elevated *p*CO_2_, while in other corals, tissue carbohydrates, proteins and lipids decline [21]. Despite mixed *p*CO_2_ effects (less than 2000 µatm) on coral respiration and photosynthesis [18,33–35], multiple lines of evidence indicate high *p*CO_2_ can affect resource allocation [20], anabolic and catabolic processes [36], and gene expression in corals indicative of changing metabolic demands [30,35]. For instance, elevated *p*CO_2_ can increase photosynthetic and heterotrophic energy acquisition [13,18,37], and may also alter the allocation of resources to growth (e.g. tissue and skeletal) or maintenance [29,38]. Such changes in resource acquisition or allocation may, therefore, influence biomass quantity [31] and composition [19,21] with concomitant consequences for coral physiology. However, OA effects on coral biomass observed to date appear complex and nonlinear [19,21,31], and effects vary (i.e. positive, negative or null effects) with light availability [20] and across species [19,21]. Considering the importance of tissue biomass to coral performance, the uncertainty of OA effects on coral biomass represents a significant knowledge gap that we aim to address here.

We tested the effects of *p*CO_2_ and light on the calcification, tissue biomass (total biomass, lipids, carbohydrates, proteins), energy equivalents (kJ or energy content) and densities of *Symbiodinium* and chlorophylls (*a* and *c_2_*) in the coral *Pocillopora acuta* (Lamarck, 1816) [39]. We address the following questions: (1) Does elevated *p*CO_2_ affect calcification, coral biomass and tissue energy content, *Symbiodinium* density and chlorophyll concentration? (2) Are the effects of *p*CO_2_ on coral biomass and calcification modulated by light availability? We reasoned high *p*CO_2_ effects on energy reserves and calcification would be attenuated by increased light availability [18] due to stimulatory effects of light on coral tissue and skeletal growth [4,16,40]. We also normalized energy reserves and calcification at two levels [41]—the surface area of the skeleton and the quantity of tissue biomass—to evaluate the scale at which these responses were affected by *p*CO_2_ and light.

## 2. Material and methods

### 2.1. Experimental design

Four experimental treatments of low and high light (LL and HL) fully crossed with ambient and high *p*CO_2_ (ACO_2_ and HCO_2_) were produced in 24 flow-through aquaria (45 l; Aqualogic, Inc., USA) (*n* = 6 tanks treatment^−1^) receiving sand-filtered natural seawater (approx. greater than 100 µm) and maintained at seasonally ambient seawater temperatures (24.94°C ± 0.05) (mean ± s.e., *n* = 680). *p*CO_2_ treatments reflected ambient Kāne‘ohe Bay seawater (ACO_2_; approx. 440 µatm *p*CO_2_), and elevated levels (HCO_2_; approx. 900 µatm *p*CO_2_) projected for the end of the century (RCP 6.0) [3]. Light treatments were programmed to a ramping 12 L : 12 D diel cycle that contrasted high light (HL; 800 µmol photons m^−2^ s^−1^ daily maximum) and 50% light attenuation conditions (LL; 400 µmol photons m^−2^ s^−1^ daily maximum) equivalent to 15.7 and 7.5 mol photons m^−2^ d^−1^.

*p*CO_2_ treatments were maintained by bubbling either ambient air (i.e. ACO_2_) or CO_2_-enriched air (i.e. HCO_2_) into four header tanks (*n* *=* 2 header tanks per *p*CO_2_ treatment). *p*CO_2_ in each header tank was controlled by a pH-stat system (Apex AquaController, Neptune Systems, USA) that dynamically regulated the flow of air or CO_2_ gas through a solenoid based on a static set-point for each seawater treatment (ACO_2_ or HCO_2_). Seawater in each header tank was delivered to six flow-through treatment tanks at approximately 1.5 l min^−1^. Seawater temperature, salinity, pH_T_ (pH on the total scale) and total alkalinity (*A*_T_) were measured in all tanks every third day of the experiment. Seawater *A*_T_ was measured by titrations, performed according to standard operating procedure 3b [42] using certified acid titrant and reference materials (see electronic supplementary material). Final values for seawater carbonate chemistry were calculated using the *seacarb* package [43] in R [44]. Further details regarding methods used to maintain experimental treatments can be found in the electronic supplementary material.

### 2.2. Coral collection

Seven adult colonies of *P. acuta* were collected on 13 and 29 October 2014 at approximately 1 m from windward facing reefs of Moku o Lo‘e (Coconut Island) in Kāne‘ohe Bay on the island of O‘ahu, Hawai‘i, USA (21°26′08.9′′ N, 157°47′12.0′′ W). Twenty-four ramets (less than or equal to 4 cm height) from each coral colony were attached to PVC-bases with Z-spar (A-788) and hot-glue, and allowed to recover for 3–5 weeks in outdoor flow-through tanks (1300 l) under attenuated natural sunlight (less than or equal to 6 mol photons m^−2^ d^−1^) receiving sand-filtered seawater and maintained at 26.05°C ± 0.01 (mean ± s.e., *n* = 4869) using a chiller (Model MT3, Aqualogic, Inc.). Subsequently, one fragment from each of the seven colonies was assigned to each of the 24 indoor treatment aquaria (*n* = 7 colony fragments tank^−1^) and allowed to acclimatize for 25 d to treatment irradiances (7.5 and 15.7 mol photons m^−2^ d^−1^), acclimatization-period temperatures 25.73 ± 0.03°C (mean ± s.e., *n* = 192), progressively increasing *p*CO_2_ (for HCO_2_ tanks), and flow. Supplemental heterotrophic feedings were not provided during acclimatization or experimental periods; however, corals had access to heterotrophic food sources in the form of microbes, dissolved organic matter and less than 100 µm plankters. Corals were exposed to *p*CO_2_ and light treatments for 32 d from 16 December 2014 to 16 January 2015 and frozen (−80°C) until further processing.

### 2.3. Physiological parameters

All coral fragments (*n* = 7 tank^−1^) were analysed for net calcification, photopigment densities, carbohydrates, proteins and total biomass. Quantification of *Symbiodinium* cell densities, lipid biomass and tissue energy content was performed on four fragments in each tank. Additional details regarding the measurement of physiological responses can be found in the electronic supplementary material. Net calcification was determined by the change in buoyant weight [45] (converted to dry weight using a density of aragonite of 2.93 g cm^−3^) and standardized to both skeletal surface area determined by wax dipping [46] and coral biomass determined by ash-free dry weight (AFDW). To quantify tissue biomass characteristics, tissues were removed from the skeleton using an airbrush filled with filtered seawater (0.2 µm). The host and symbiont extract (hereafter, tissue slurry) was briefly homogenized, subsampled and frozen at −20°C. *Symbiodinium* densities were determined from replicate counts (*n* = 6–8) of tissue slurry on a haemocytometer and normalized to surface area. Concentrations of chlorophyll *a* and *c*_2_ were determined following [47] using 100% acetone and normalized to both surface area and symbiont cells.

Total biomass was measured from the difference in dried (60°C) and burned (4 h at 450°C) masses of an aliquot of tissue slurry, and the ash-free dry weight of biomass was expressed as mg biomass cm^−2^. Total soluble lipid biomass (hereafter, lipids) was extracted from freeze-dried tissues (host + symbionts) in 2 : 1 chloroform : methanol, following [19] and carbohydrate and total protein (soluble + insoluble) biomass were quantified on subsamples of the tissue slurry using established methods (see electronic supplementary material for further details). The equivalent energetic value of biomass (i.e. energetic content) was determined by summing the specific enthalpy of combustion (kJ g^−1^) for lipid, carbohydrate and protein biomass [48]. Biomass energy reserves (lipids, carbohydrates, proteins) and energy content were each normalized to skeletal surface area and tissue AFDW.

### 2.4. Statistical analyses

Studies of coral physiology commonly standardize response variables to either skeletal area or biomass units (e.g. dry weight, protein) [41]. In scleractinian corals, tissue biomass can vary across the surface of individual coral colonies [49] and among colonies differing in size [38]. In some cases, normalizing physiological metrics to a quantity reflecting the amount of live material (i.e. biomass) may be preferable [41] in order to account for effects of colony size or if metrics are not rate-limited by metabolite flux across coral tissues (e.g. respiration, photosynthesis). However, the mass of tissue energy reserves has been normalized to skeletal surface area [38], and sometimes to biomass [25], with one recent outcome being that the trends as a function of *p*CO_2_ treatment conditions are inconsistent [19,21]. In order to evaluate treatment effects on coral biomass and calcification, and address the potential role of normalization (i.e. surface area versus grams of dry weight) in the interpretation of treatment effects, we took the following approach. First, we tested the broad hypothesis that corals responded to treatments by using a multivariate principal component (PC) analysis that included coral calcification and biomass metrics normalized to either surface area or biomass. This approach provided a test of the overall treatment effect without inflated Type I error rate. Second, to evaluate which variables were most influential in driving multivariate effects, we applied univariate hypothesis tests on individual metrics to determine where treatment effects existed (see electronic supplementary material for a detailed description of statistical tests).

Analyses of seawater carbonate chemistry among replicate treatment tanks were examined using separate one-way ANOVAs with tank as a predictor and *p*CO_2_, pH_T_ and *A*_T_ as explanatory variables. *p*CO_2_ and light effects on biological response variables and multivariate PCs were analysed using a linear mixed-effect model in the *lme4* package in R [50]. *p*CO_2_ and light treatments were treated as fixed effects, colony as a random effect, and tank as a random effect nested within *p*CO_2_ × light treatment. Correlations between multivariate PCs and response variables were tested using Pearson's correlation coefficient using cor.test in R. All analyses were performed using R v. 3.2.1 [44]. Raw data and code to reproduce this work is archived at Dryad (http://dx.doi.org/10.5061/dryad.5vg70) [51].

## 3. Results

### 3.1. Treatment conditions

Experimental treatments were precisely regulated at target levels (table 1). Mechanical issues in two replicate HL–HCO_2_ tanks towards the end of the experiment led to the *a priori* removal of these tanks and constituent corals from further analyses. Therefore, final replication for HL–HCO_2_ treatments was four tanks per treatment and for all other treatments, six. Corals were maintained under mean *p*CO_2_ treatments of 435 ± 8 µatm *p*CO_2_ (ACO_2_) and 957 ± 30 µatm *p*CO_2_ (HCO_2_) equivalent to a pH_T_ of 8.00 ± 0.01 and 7.71 ± 0.01 (±s.e., *n* = 84 and 69) (table 1). Seawater treatments differed in *p*CO_2_ (*p <* 0.001) and pH_T_ conditions (*p *< 0.001) and *A*_T_ was not affected by CO_2_ treatment (*p* = 0.110). *p*CO_2_ and pH_T_ did not differ among replicate CO_2_ treatment tanks (*p* ≥ 0.060).

**Table 1.** Summary of environmental conditions in the experimental treatment tanks between 16 December 2014 and 16 January 2015. Seawater *A*_T_, pH on the total scale (pH_T_), temperature (Temp) and salinity (approx. 34.3) were used to calculate the partial pressure of carbon dioxide (*p*CO_2_), concentrations of dissolved inorganic carbon species, and the aragonite saturation state (Ω_arag_) using the package *seacarb* in R. LL–ACO_2_ = low light–ambient *p*CO_2_; LL–HCO_2_ = low light–high *p*CO_2_; HL–ACO_2_ = high light–ambient *p*CO_2_; HL–HCO_2_ = high light–high *p*CO_2_; PAR = photosynthetically active radiation, integrated over 12 h (mol photons m^−2^ d^−1^); *n* *=* 6 replicate tanks treatment^−1^, except HL–HCO_2_ *n* = 4 replicate tanks. Values are mean ± s.e (*n*).

treatment	PAR	pH_T_	*A*_T_ (μmol kg^−1^)	*p*CO_2_ (μatm)	${\text{HCO}}_{3}^{-}$ (μmol kg^−1^)	${\text{CO}}_{3}^{2-}$ (μmol kg^−1^)	Ω_arag_
LL–ACO_2_	7.5	7.99 ± 0.01 (42)	2177 ± 3 (42)	451 ± 11 (42)	1733 ± 8 (42)	179 ± 3 (42)	2.84 ± 0.06 (42)
LL–HCO_2_	7.5	7.71 ± 0.02 (41)	2184 ± 4 (41)	957 ± 39 (41)	1917 ± 12 (41)	108 ± 5 (41)	1.72 ± 0.07 (41)
HL–ACO_2_	15.7	8.01 ± 0.01 (42)	2179 ± 3 (42)	420 ± 11 (42)	1714 ± 9 (42)	187 ± 4 (42)	2.97 ± 0.06 (42)
HL–HCO_2_	15.7	7.71 ± 0.02 (28)	2184 ± 4 (28)	957 ± 47 (28)	1920 ± 12 (28)	106 ± 5 (28)	1.69 ± 0.08 (28)

### 3.2. Multivariate response analysis

**Figure 1.**
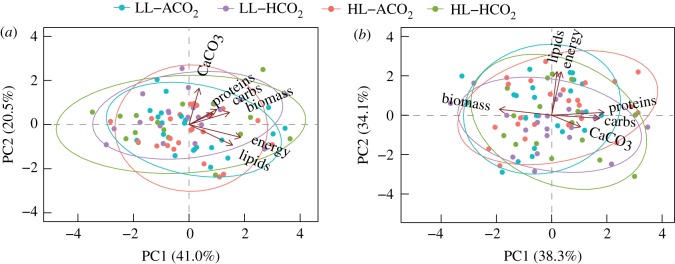
Principal component analyses (PCA) for energy reserves and net calcification normalized to (*a*) skeletal surface area (cm^−2^) and (*b*) tissue biomass (gdw^−1^), with total biomass (mg AFDW cm^−2^) present in each data matrix. Axis values in parentheses represent proportion of total variance associated with the respective PC. Arrows represent correlation vectors for response variables, and ellipses represent 90% point density according to treatments. Treatment details can be found in table 1.

Complete outputs from all statistical models can be found in electronic supplementary material, tables S2–S4; summarized model outputs are displayed in table 2. Two principal components with eigenvalues greater than 1.0 explained 62% and 72% of observed variance for area- and biomass-normalized variables, respectively (table 2; electronic supplementary material, table S2). Graphical inspection of PC-biplots for area-normalized responses showed poor separation according to experimental treatments (figure 1*a*), and PC1 and PC2 were not affected by light or *p*CO_2_ (*p* *≥* 0.114) (table 2; electronic supplementary material, table S2). Area-normalized PC1 (41.0% variance explained) was positively correlated with all responses (*p *< 0.001), except calcification (*p* = 0.105). PC2 negatively correlated with lipids and energy content (*p* ≤ 0.008) and positively correlated with all other metrics (*p* ≤ 0.019). Conversely, PC-biplots for biomass-normalized responses showed the greatest degree of divergence between ambient and high *p*CO_2_ treatments along PC2 (figure 1*b*), and PC2 was affected by CO_2_ treatment (*p* = 0.028) (table 2). PC1 was not affected by light or *p*CO_2_ (*p* *≥* 0.269) (table 2). Biomass-normalized PC2 was positively correlated with lipids and tissue energy content (*p* < 0.001), and negatively correlated with calcification (*p* = 0.015) (figure 1*b*). Hence, elevated *p*CO_2_ conditions had significant effects on corals when skeletal and biomass energy reserve metrics were normalized to tissue biomass, and *p*CO_2_ treatments best explained the opposing relationship of biomass quality (lipids, energy content) and calcification.

**Table 2.** Summary of *p*-values for *p*CO_2_ and light effects on PC loadings and response variables normalized to skeletal area and tissue biomass. Summarized output from linear mixed-effect models; full models can be found in the electronic supplementary material. PC, principal component; bold *p*-values represent significant effects less than 0.05; M dashes are present where responses were not measured.

	area-normalized (cm^−2^)			biomass-normalized (gdw^−1^)		
	effect			effect		
response variables	*p*CO_2_	light	*p*CO_2_ × light	*p*CO_2_	light	*p*CO_2_ × light
Multivariate models						
PC1	0.493	0.624	0.856	0.689	0.269	0.777
PC2	0.114	0.562	0.359	**0**.**028**	0.718	0.919
Univariate models						
calcification	0.605	0.793	0.861	0.586	0.277	0.879
total biomass	0.950	0.210	0.677	—	—	—
proteins	0.270	**0**.**010**	**0**.**038**	0.415	0.702	0.492
carbohydrates	0.351	0.505	0.132	0.342	**0**.**040**	0.297
lipids	0.145	0.751	0.683	**0**.**040**	0.436	0.917
energy content	0.201	0.543	0.891	**0**.**041**	0.445	0.952
*Symbiodinium* cells	0.338	0.124	0.483	—	—	—
chlorophyll *a*	0.993	<**0**.**001**	0.144	—	—	—
chlorophyll *c*_2_	0.961	<**0**.**001**	0.114	—	—	—
chlorophyll *a* cell^−1^^a^	0.886	0.109	0.587	—	—	—
chlorophyll *c*_2_ cell^−1^^a^	0.765	0.217	0.449	—	—	—

^a^Photopigment concentrations normalized to *Symbiodinium* cell.

### 3.3. Net calcification rates, *Symbiodinium* densities and chlorophylls

**Figure 2.**
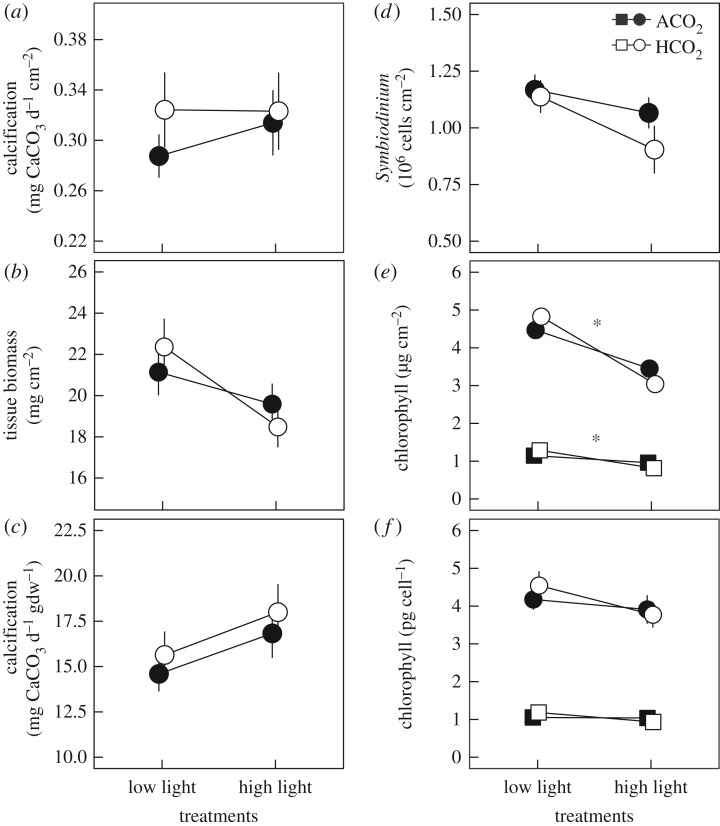
Net calcification, total biomass, photopigment concentrations and *Symbiodinium* densities of *P. acuta* corals exposed to light treatments (7.5 and 15.7 mol photons m^−2^ d^−1^) and ambient *p*CO_2_ (ACO_2_) and high *p*CO_2_ (HCO_2_) (table 1). (*a*) Area-normalized net calcification rates, (*b*) total tissue biomass, (*c*) biomass-normalized net calcification rates, (*d*) *Symbiodinium* densities and (*e*) chlorophyll *a* (circles) and chlorophyll *c*_2_ (squares) densities normalized to skeletal area and (*f*) symbiont cells. Values displayed are means ± s.e.; *n* = 28 (HL–HCO_2_) and *n* = 39–41 (all other treatments), except (*d*,*f*) *n* = 16 (HL–HCO_2_) and *n* = 24 (all other treatments). Asterisks indicate a statistical difference (*p* < 0.05) between light treatments.

*p*CO_2_ and light treatments had no effect on net calcification rates normalized to skeletal area (*p* ≥ 0.605; figure 2*a*) or biomass (*p* ≥ 0.210; figure 2*c*) (table 2; electronic supplementary material, tables S3 and S4). However, biomass-normalized calcification tended to be 15% higher at high light relative to low light conditions. *Symbiodinium* density cm^−2^ was not affected by treatments (*p* ≥ 0.124) (table 2; figure 2*d*), but chlorophyll *a* and *c*_2_ cm^−2^ declined by 28% and 25% at high light relative to low light treatments (*p *< 0.001) (table 2; figure 2*e*). However, photopigment concentrations per *Symbiodinium* cell were not affected by treatments (*p* ≥ 0.109) but tended to be lower under high light conditions (table 2; figure 2*f*).

### 3.4. Tissue energy reserves and normalization approaches

**Figure 3.**
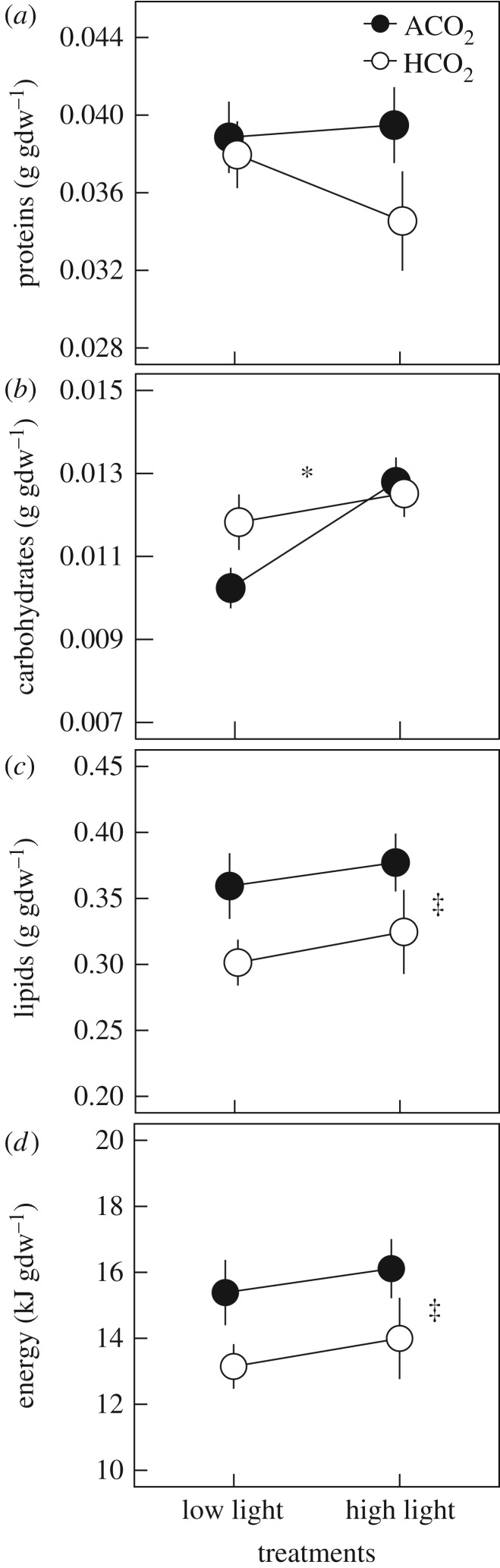
Biomass-normalized (gdw^−1^) (*a*) proteins, (*b*) carbohydrates, (*c*) lipids and (*d*) tissue energy content in *P. acuta* corals exposed to light treatments (7.5 and 15.7 mol photons m^−2^ d^−1^) and ambient *p*CO_2_ (ACO_2_) and high *p*CO_2_ (HCO_2_) (table 1). Values displayed are means ± s.e.; *n* = 16–24 for lipid biomass and energy content, for other variables *n* = 28 (HL–HCO_2_) and *n* = 41–42 (all other treatments). Symbols indicate statistical differences (*p* < 0.05) between light (*) or *p*CO_2_ (‡) treatments.

Treatments had no effect on total biomass cm^−2^ (*p* ≥ 0.210) (table 2; figure 2*b*) or protein per gram of dry coral tissue (gdw^−1^) (*p* ≥ 0.415) (table 2; figure 3*a*). Carbohydrate gdw^−1^ increased 15% in corals at high light relative to low light conditions (*p* = 0.040) (figure 3*b*), and corals exposed to 957 µatm *p*CO_2_ had 15% less lipid gdw^−1^ (*p* = 0.040) (figure 3*c*) and 14% less biomass energy content gdw^−1^ (*p* = 0.041) than corals at 435 µatm *p*CO_2_ (figure 3*d*) (table 2; electronic supplementary material, table S4).

The effects of treatments on area-normalized energy reserves differed from effects on biomass-normalized energy reserves. No effect of *p*CO_2_, light or their interaction was observed for carbohydrate cm^−2^, lipid biomass cm^−2^ or tissue energy content cm^−2^ (*p* ≥ 0.132) (table 2; electronic supplementary material, figure S1*b*–*d* and table S3). However, protein biomass cm^−2^ was affected by the interaction of *p*CO_2_ × light (*p* = 0.038) and light (*p* *=* 0.010) but not *p*CO_2_ alone (*p* = 0.270) (table 2; electronic supplementary material, figure S1*a* and table S3). Mean protein (mg cm^−2^) was 17–23% lower at HL–HCO_2_ relative to other treatments (post hoc: *p* ≤ 0.017) but was not significantly different from the HL–ACO_2_ treatment (post hoc: *p* = 0.157) (electronic supplementary material, figure S1*a*).

## 4. Discussion

### 4.1. Ocean acidification and light effects on calcification

Our results demonstrate calcification in *P. acuta* was not affected by *p*CO_2_ (435 and 957 µatm) or light availability (7.5 versus 15.7 mol photons m^−2^ d^−1^). The lack of an effect of *p*CO_2_ on calcification contrasts with the majority of studies showing OA reduces calcification rates in corals and other marine calcifiers [5,6], but is consistent with previous work showing net calcification in *Pocillopora* spp. is insensitive to elevated *p*CO_2_ (less than or equal to 1970 µatm *p*CO_2_) [19,52,53] (but see [11]). Corals from Kāne‘ohe Bay experience significant diel variability in *p*CO_2_ [54] and have been hypothesized to exhibit varying degrees of acclimatization or local adaptation to high *p*CO_2_. However, a pan-Pacific collection (including Kāne‘ohe Bay) of the congener *Pocillopora damicornis* revealed this species was resistant to elevated *p*CO_2_ effects on calcification across geographical locations [53]. This finding suggests *p*CO_2_ history alone does not completely explain the resistance of *Pocillopora* spp. calcification to OA, but rather a combination of physiological and/or genetic factors may also underpin OA resistance in *P. acuta* and related pocilloporids.

The interactive effects of *p*CO_2_ and light on coral calcification varies among coral species [18,55] and life-history stages [9,32], and may depend on the mechanism and/or rate by which species calcify [52,56,57] as well as their capacity to regulate internal pH [58–61]. While light availability modulates OA effects on calcification in some corals [9,17,18], meta-analysis reveals the heterogeneous response of coral calcification to declining Ω_arag_ is not well explained by light intensity [6]. The absence of *p*CO_2_ or light effects on *P. acuta* calcification in the current study has also been reported in other corals. For instance, *Porites rus* calcification was similarly unaffected by *p*CO_2_ (400 versus 700 µatm) at 6.2 and 28.7 mol photons m^−2^ d^−1^ [32], and light availability (3.5–30.2 mol photons m^−2^ d^−1^) did not influence the response of *Porites compressa* to decreasing Ω_arag_ (2.48 versus 5.05) [62] (electronic supplementary material, table S1). In part, the observation in some corals of light intensity mitigating OA effects on calcification may be linked to light-dependent usage of dissolved inorganic carbon substrates (e.g. ${\text{HCO}}_{3}^{-}$ or ${{\text{CO}}_{3}}^{2-}$) in calcification [57] and/or stimulatory effects of light availability on *Symbiodinium* photosynthesis, coral metabolism, ion regulation and the synthesis of organic matrix at the calcifying surface [4,63,64]. In the present study, the lack of *p*CO_2_ effects on *P. acuta* calcification at both light treatments suggests beneficial effects of light availability on coral performance [18] were realized at both light-saturating treatments (7.5 versus 15.7 mol photons m^−2^ d^−1^), or this coral species possesses mechanisms enabling it to maintain comparable rates of calcification at both 435 and 957 µatm *p*CO_2_, potentially through pH regulation at the site of calcification [65].

The sensitivity of coral calcification to OA may reflect the differential capacity of coral species to upregulate extracellular pH in the calcifying fluid at the site of calcification [7,60,65,66]. Ca^2+^/H^+^ ATPases exchange ions across the calicoblastic epithelia to produce locally high pH in the calcifying fluid (approx. 0.5–2.0 pH units above external seawater) [60,66,67]. Alkalinization of the calcifying fluid shifts the chemical equilibrium of dissolved inorganic carbon in favour of ${{\text{CO}}_{3}}^{2-}$ and facilitates the diffusion of molecular CO_2_ into the calcifying fluid [59], thereby increasing [DIC] and Ω_arag_ (i.e. 15–22) and promoting the precipitation of aragonite [7,60]. Under OA, a higher H^+^ concentration in seawater may challenge the capacity for corals to export H^+^ from tissues [68], which is hypothesized to increase the metabolic costs of upregulating calcifying fluid pH and Ω_arag_ and cause reductions in CaCO_3_ precipitation rates [67]. On the other hand, corals can compensate for declining Ω_arag_ in the calcifying fluid by increasing the incorporation of organic matrix proteins into the skeleton [69] which act to increase the nucleation of aragonite crystals [70]. A more organic-rich skeleton may reduce the sensitivity of corals (and other marine calcifiers) to OA by reducing the free energy required for calcification [71], although the synthesis of organic skeletal material requires significantly more energy than inorganic CaCO_3_ production [72] and additional energy inputs may be necessary. In corals, calcification accounts for 30% of energy demand [28]. Thus, thermodynamically unfavourable conditions (low Ω_arag_) causing greater energetic expenditures for calcifying fluid regulation and/or organic matrix synthesis [73] may additively influence the capacity of corals to maintain high calcification rates, or otherwise impact their energy balance, under OA.

### 4.2. Ocean acidification and light effects on coral biomass

In agreement with previous laboratory and field studies [33,74] (but see [75]), elevated *p*CO_2_ did not lead to coral bleaching or reductions in symbiont densities and/or chlorophyll concentration in low or high light treatments. Instead, corals photoacclimatized [76] to increasing light levels by reducing concentrations of chlorophylls (*a* and *c_2_* cm^−2^), although without appreciable loss of symbiont cells. However, exposure to 957 µatm *p*CO_2_ altered the composition of *P. acuta* biomass relative to corals maintained at 435 µatm *p*CO_2_ regardless of light conditions. Declining lipid biomass at high *p*CO_2_ suggests that lipid reserves were either catabolized to meet energetic demands [30] and/or lipid precursors were allocated to processes other than the formation of lipid biomass. Under OA conditions corals may require greater energy investments in the process of calcification in order to maintain high rates of aragonite precipitation [28,73]. For instance, greater energy inputs from dissolved nutrients [77] and heterotrophic feeding [12] can lessen negative effects of high *p*CO_2_ (less than or equal to 830 µatm) on calcification in some corals. While heterotrophic food sources available to corals in the present study were restricted (less than 100 µm, sand-filtrated seawater), it is likely that natural nutrient sources in seawater (e.g. dissolved inorganic and organic nutrients, pico- and nanoplankton, small zooplankton) supplemented symbiont-derived nutrition [78]. The ability for corals to increase heterotrophic feeding in response to changes in photoautotrophic nutrition or energy demand contributes to physiological resilience [79], yet the capacity for many corals, including *P. acuta,* to be nutritionally flexible under normal and stressed physiological states has yet to be quantified. Recent evidence suggests some corals may increase rates of heterotrophic feeding in response to elevated *p*CO_2_ [13]. However, *in situ* elevated *p*CO_2_ reduces the abundance of zooplankton on corals reefs [80] and may reduce heterotrophic nutrition and/or increase metabolic costs associated with prey capture. Therefore, while a combination of zooplanktivory and biomass catabolism may be employed by corals as an acclimatization response to physiological stress [25,79]—including elevated *p*CO_2_ [13]—OA effects on coral biomass (this study) and zooplankton availability [80] may negatively impact coral performance and their response to physiological challenges [27,81,82].

In corals, tissue growth is sensitive to changing resource availability and physiological stress [16,22,38]. Under these conditions, skeletal growth may come at the expense of reduced tissue growth [38] and biomass may be broken down to support metabolism [25]. Consistent with this hypothesis are observations that low pH (7.4–7.7) causes an upregulation of coral genes involved in lipolysis and β-oxidation pathways, suggesting tissue reorganization and the catabolism of fatty acid reserves [30,35]. Such changes in gene expression could explain the reduction in lipid biomass observed here, as well as the negative relationship between elevated *p*CO_2_ and coral tissue biomass (*Pocillopora damicornis*, [31]) and lipids cm^−2^ (*Acropora millepora*, *Montipora monasteriata* [21]). By contrast, *Porites rus* and *Acropora pulchra* tissue biomass [20,31] and *A. millepora* and *P. damicornis* lipids gdw^−1^ [19] displayed a positive parabolic relationship with elevated *p*CO_2_. These effects may be explained by elevated [DIC] stimulating *Symbiodinium* productivity and carbon translocation [18,37,83] with downstream effects on biomass synthesis. Alternatively, supplemental heterotrophic feedings [19] may overcome OA-induced energy deficits and replenish lipid reserves [13]. Together, these examples illustrate that *p*CO_2_ is likely to have nonlinear and heterogeneous effects on coral biomass, as has been noted for OA effects on calcification [6,52]. Nonetheless, our finding that lipid biomass and energy content gdw^−1^ declined in *P. acuta* following one month at 957 µatm *p*CO_2_ supports the hypothesis that OA affects energetic requirements in corals, potentially related to metabolic costs or the acquisition and allocation of resources.

At the organismal level, elevated *p*CO_2_ (less than 2000 µatm) has negligible effects on aerobic respiration [34]; however, elevated *p*CO_2_ can elicit compensatory changes at the cellular level that affect energy allocation, gene expression and physiological resilience [30,35]. For instance, sea urchin larvae responded to OA with a 30% increase in the metabolic energy allocated to protein synthesis and ion transport [29]. Such flexibility in energy allocation may be critical for organisms to respond to environmental stress when metabolic demands exceed metabolic capacity. In the present study, it is uncertain whether longer duration exposures to 957 µatm *p*CO_2_ would result in further reductions (or stabilization) of *P. acuta* lipid biomass and eventually cause skeletal and biomass growth to decline. In any case, decreased biomass quality may have wide-reaching effects on coral performance, including the susceptibility to post-bleaching mortality and reproduction [23,25,38]. Therefore, unravelling the long-term consequences of OA on biomass energetics at the organismal and cellular level should be a priority for future research.

Previous studies have observed mixed responses of total biomass to high *p*CO_2_. For example, biomass was not affected by *p*CO_2_ (less than or equal to 741 µatm) in four Indo-Pacific corals (including *P. damicornis*) [19], and *P. rus* total biomass at two irradiances was insensitive to changes in *p*CO_2_ (less than or equal to 1100 µatm) [84]. However, high *p*CO_2_ has been shown to increase total biomass in some coral species when maintained under high light conditions [20,31]. In the present study, total biomass (mg AFDW cm^−2^) was not affected by treatments, yet area-normalized protein (a common proxy for biomass; [41]) was reduced approximately 20% under 957 µatm *p*CO_2_ and 15.7 mol photons m^−2^ d^−1^. Together, high light and high *p*CO_2_ may interfere with aspects of protein metabolism [36] or turnover [29] in *P. acuta* manifesting in reduced protein per skeletal surface [21,85]. However, in our study the total organic fraction of *P. acuta* biomass (i.e. AFDW cm^−2^) appears less sensitive to *p*CO_2_ and light effects, potentially due to dynamic changes in the concentration of other tissue macromolecules aside from proteins.

Finally, the interpretation of responses to OA effects was dependent on the approach used to normalize response variables. Multivariate tests on biomass-normalized responses revealed significant effects of *p*CO_2_ on *P. acuta* with an opposing relationship between net calcification rates and biomass quantity and quality (i.e. per cent lipid and energy content). This finding was supported by univariate tests where *p*CO_2_ reduced biomass lipid and energy content. Conversely, *p*CO_2_ did not affect responses normalized to skeletal area (except for protein biomass). Area- and biomass-normalizations are often used interchangeably, yet these normalizations are not equivalent due to allometric growth in corals and variability in the quality and quantity of tissue biomass over the coral skeleton [38,49]. Such factors may confound area-normalized physiological responses not directly related to skeletal area [41]. Indeed, the differences observed here between area- and biomass-normalized metrics suggest disparate trends in *p*CO_2_ effects on biomass observed in other studies may in part reflect normalization approaches [19,21] and/or sampling techniques (e.g. tip subsampling versus whole fragment tissues). We recommend future studies consider the significance of normalization approaches in representing physiological data [41,86], and suggest that energy reserve-specific metrics be normalized to biological units (i.e. living tissue biomass) so that the physiological implications of environmental change on coral tissues may be clarified without the potential confounding effects of skeletal area.

## 5. Conclusion

This study demonstrates that one-month exposure to OA conditions predicted for the year 2100 did not affect *Pocillopora acuta* calcification rates, but elevated *p*CO_2_ reduced lipid biomass gdw^−1^ and energy content gdw^−1^ and interacted with high light to reduce protein cm^−2^. Considering the significance of lipid biomass for coral performance (e.g. post-stress physiology, reproduction), reduction in lipid biomass (and biomass energy content) may negatively affect *P. acuta* and reduce its physiological resilience to rising seawater temperatures. Our findings raise a testable hypothesis for *P. acuta*: that maintenance of present-day calcification rates under OA incurs an energetic cost, which is met through catabolism of, or diversion of energy that otherwise would have been stored as, tissue lipids. Finally, we report the interpretation of *p*CO_2_ effects on tissue biomass were dependent on whether energy reserves were normalized to tissue biomass or skeletal area. We propose data normalization to be an overlooked aspect of coral physiology that may be contributing to the observed variance in OA effects on corals.

## Supplementary Material

Supplemental Methods, Additional Tables and Figures

## Data accessibility

All data and R code to reproduce figures and analyses are available on Dryad (doi.org/10.5061/dryad.5vg70) [51].
