# Supplementary material for: Elevated pCO2 affects tissue biomass composition, but not calcification, in a reef coral under two light regimes
Source: R Soc Open Sci. 2017 Nov 1;4(11):170683. doi: 10.1098/rsos.170683 (PMC5717633; doi:10.1098/rsos.170683)
Supplement: Supplemental Methods, Additional Tables and Figures [file rsos170683supp1.docx]

**Wall et al.: Elevated pCO_2_ affects tissue biomass composition, but not calcification, in a reef coral under two light regimes**

**Supplemental materials**

**1. Additional methods**

(a) *Taxonomic identification*

Coral samples were identified as *Pocillopora acuta* rather than the morphologically similar *P. damicornis* [1]. Our laboratory has performed molecular identifications of pocilloporid colonies at Moku o Lo‘e Island (Hawai‘i Institute of Marine Biology, HIMB) and within the larger Kāne‘ohe Bay reef system that revealed that *P. acuta* is overwhelmingly the dominant coral of the two species at our sampling location. We also consulted several scientists at HIMB regarding species identifications at our collection site.

(b) *Experimental treatments*

Light treatments were created by suspending a 75 W light emitting diode module over each tank (AI Sol White, Blue, Royal Blue; Aqua Illuminations, USA), calibrated with a 4π quantum sensor (LI-193, Li-Cor, USA) connected to an LI-1400 light meter (Li-Cor). Lights were programmed to increase each day from 0500 – 1000 hrs, sustain maximum (400 or 800 μmol photons m^-2^ s^-1^) for 2 h, and decrease to darkness by 1700 hrs, resulting in a 12h light : 12h dark diel cycle. These light treatments are ecologically relevant to reef corals on Kāne‘ohe Bay patch reefs, where daily integrated light intensities at 1 m depth near our collection site range from 10 – 20 mol photons m^-2^ d^-1^ and ~ 300 – 1,100 μmol photons m^-2^ s^-1^ maximum daily irradiance for the period of November – January [2].

Seawater temperature, salinity, pH_T_ (pH on total scale) and total alkalinity (*A_T_*) were measured in all tanks two times per week.  Seawater temperature (24.59˚C ± 0.06) (mean ± SE, *n* = 153) during the 32 d experimental period was independently maintained in each treatment tank using digital temperature controllers (Model TR115DN; Aqualogic Inc., USA) and submersible heaters. Temperature in tanks was monitored using a certified digital thermometer (5-077-8, +/- 0.05°C, Control Company, USA), and the salinity of incoming seawater (ca. 34 salinity) was monitored using a conductivity meter (YSI 63, YSI Inc., USA); pH_T_ was measured using a benchtop pH meter (Orion 3-Star, Thermo Fisher Scientific, USA) and pH probe (DG115-SC, Mettler-Toledo, LLC, USA) calibrated against certified Tris standard at a range of temperatures (Dickson Lab, UCSD) [3]. Titrations were performed using an open-cell, potentiometric automatic titrator (T50, Metler-Toledo, USA) filled with certified acid titrant (Dickson Lab, UCSD).  Titrations of certified reference materials of known *A_T_* (Batch 137 and 140) provided by A.G. Dickson (UCSD) were titrated prior to and alongside treatment seawater titrations, with our analyses differing on average < 0.8% or 17 μmol kg^-1^ (*n*= 21) from certified values.

(c) *Physiological parameters*

The concentration of chlorophyll *a*and *c_2_* was quantified following a modified protocol from [4]. An aliquot of homogenized tissue slurry (1 ml) was centrifuged (1,600 × g for 3 min), pelleting symbiont cells. The supernatant was decanted and 1 ml of 100% acetone was added to the pellet and allowed to incubate in darkness at -20 °C for 36 h. Chlorophyll concentrations were calculated using trichromatic equations for dinoflagellates [5]. Total lipid biomass (hereafter, lipids) was measured by lyophilizing a subsample of the coral slurry (host + symbiont) for 12 h, and extracting lipids from the freeze-dried tissue in 2:1 chloroform:methanol, following [6]. The lipid extract was filtered through a GF/F filter (0.7 μm), washed with 0.88% KCl, followed by 100% chloroform and 0.88% KCl washes, evaporated to dryness under nitrogen gas (5.0 purity grade), and quantified gravimetrically on a microbalance. Carbohydrates were determined spectrophotometrically using the phenol-sulfuric acid method with glucose as a standard [7]. Total soluble and insoluble protein (hereafter, proteins) was determined by adding 0.1 M NaOH to the tissue slurry, heating (90 °C for 1 h), and using the bicinchoninic acid method (Pierce BCA Protein Assay Kit, Thermo Fisher Scientific) with a bovine serum albumin standard [8]. The energy content of coral tissues was calculated using the specific enthalpy of combustions for lipids (−39.5 kJ g^−1^), proteins (−23.9 kJ g^−1^), and carbohydrates (−17.5 kJ g^−1^) [9].

(d) *Statistical analysis*

A principal component analysis (PCA) using a scaled and centered correlation matrix was used to test the relationship among net calcification, total biomass, tissue reserves and energy content among data normalization approach (area-normalized vs. biomass-normalized response variables) and experimental treatments. The PCA data matrix included those fragments where all tissue biomass metrics and calcification had been measured (*n* = 4 fragments tank^-1^); total biomass from AFDW (mg cm^-2^) was included in both data matrices. The multivariate relationship between the two principal components (PC) explaining the greatest variance (PC1 and PC2) was graphically examined for area- and biomass-normalized response variables. Correlations between PCs and response variables were tested using Pearson's correlation coefficient using cor.test in R. To interpret treatments effects on PCs, component loadings with eigenvalues > 1.0 were tested to meet assumptions of ANOVA and examined using linear mixed effect models (*see below*).

Linear mixed-effect models were performed using the *lme4* package in R [10]. Random effects for colony (1|Colony) and replicate tank nested in pCO_2_-light treatment (1|Treatment:Tank) were included in a complete model with fixed effects (pCO_2_ and Light). The decision to retain or exclude random effects in models was determined by sequentially dropping random effects and performing likelihood ratio tests among models. Assumptions of normality and homoscedasticity of response variables and principal components were confirmed by graphical analysis of residuals; data transformations were applied when assumptions were violated. ANOVA tables were generated for fixed effects using Type II sum of squares with Satterthwaite degrees of freedom using *lmerTest* [11]. Significant interactive effects (*p <* 0.05) were examined by least-square means with a Tukey adjustment in the *lsmeans* package [12]

| **Table S1.** Summary of selected works testing pCO_2_ and light treatments on coral calcification using single irradiances (*top panel*) and multiple irradiances (*lower panel*) | | | | | | | | | |
| --- | --- | --- | --- | --- | --- | --- | --- | --- | --- |
| Species | Life stage | μatm pCO_2_ | | Daily PAR | | OA effect on growth | | Reference | |
| *pCO_2_ effects under single light level* | | | |  | |  | |  | |
| *Porites astreoides* | recruit | 480, 560, 720 | | 0.5 | | 50 – 78% decline skeletal extension | | [13] | |
| *Favia fragum* | recruit | 421, 1311 | | 2.7 | | 37% decline corallite mass | | [14] | |
| *Astrangia poculata* | adult | 390, 780 | | 3.3 | | 66% decline *G_N_* | | [15] | |
| *Acropora cervicornis* | adult | 385, 800 | | 5.8 | | 14% decline *G_N_* | | [16] | |
| *Stylophora pistillata* | adult | 385, 1904, 3970 | | 7.2 | | 18% decline *G_N_* | | [17] | |
| *Stylophora pistillata* | adult | 460, 760 | | 15.1 | | 26% decline *G_N_* | | [18] | |
| *Porites* spp. | adult | 411, 804 | | 25.9 | | no effect of OA | | [19] | |
| *Porites rus* | adult | 411, 804 | | 25.9 | | 28% decline *G_N_* | | [19] | |
| *pCO_2_ effects under multiple light levels* | | |  | |  | |  | |  |
| *Pocillopora damicornis* | recruit | 490, 900 | | 1.0, 3.5, 9.5 | | 0%, 32%, 12% decline in *G_N_*  under OA with increasing light | | [20] | |
| *Acropora millepora* | adult | 427, 1073 | | 1.5, 6.5 | | no light × pCO_2_ interaction; 48% and 144% decline in *G_N_* and *G_D_* under OA | | [21] | |
| *Porites compressa* | adult | 336, 641 | | 4.0, 6.0, 12.6, 23.3 | | 0%, 44%, 27%, and 10% decline in *G_N_* under OA with increasing light | | [22] | |
| *Acropora horrida* | adult | 390, 725 | | 4.3, 17.3 | | 50% (LL) and 10% (HL) decline in *G_L_* under OA; 40% decline in *G_D_* at LL and HL under OA | | [23] | |
| *Porites cylindrica* | adult | 390, 725 | | 4.3, 17.3 | | 80% (LL) and 50% (HL) decline in *G_L_* under OA; 80% decline in *G_D_* at LL and HL under OA | | [23] | |
| *Acropora pulchra* | adult | 400, 750, 1100 | | 4.3, 18.7 | | no effect of OA; 55% decline *G_N_*  at LL | | [24] | |
| *Porites rus* | adult | 375, 710 | | 6.2, 28.8 | | no effect of OA or PAR | | [25] | |
| OA = ocean acidification conditions of low pH, high pCO_2_, and/or low aragonite saturation state (Ω_arag_); PAR = photosynthetically active radiation; Daily PAR = mol photons m^-2^ d^-1^ integrated over the light period in the reference study; recruit = newly settled or post-settlement juvenile corals; adult = fragments collected from adult colonies; *G_N_ =* net calcification; *G_L_* = calcification in light; *G_D_* = calcification in dark; LL = low light; HL = high light. | | | | | | | | | |

**2. Additional tables**

| **Table S2.** Principal component loadings with eigenvalues > 1.0 analyzed in linear mixed effect models. | | | | | | | | | | | | | |
| --- | --- | --- | --- | --- | --- | --- | --- | --- | --- | --- | --- | --- | --- |
|  | | *Effect* | | | *SS* | | *df* | *F* | | *p* | | |  |
| Area-normalized | | |  | | |  | |  |  | | |  | |
| PC1 (41.0%) | | pCO_2_ | | | 1.004 | | 1,21 | 0.488 | 0.493 | | | | |
|  | | Light | | | 0.510 | | 1,21 | 0.248 | 0.624 | | | | |
|  | | pCO_2_ × Light | | | 0.070 | | 1,21 | 0.034 | 0.856 | | | | |
|  | |  | | |  | |  |  |  | | | | |
| PC2 (20.5%) | | pCO_2_ | | | 2.899 | | 1,22 | 2.704 | 0.114 | | | | |
|  | | Light | | | 0.372 | | 1,22 | 0.347 | 0.562 | | | | |
|  | | pCO_2_ × Light | | | 0.940 | | 1,22 | 0.877 | 0.359 | | | | |
| Biomass-normalized | | | |  | | |  | |  | |  | | |
| PC1 (38.3%) | | pCO_2_ | | | 0.279 | | 1,23 | 0.164 | 0.689 | | | | |
|  | | Light | | | 2.185 | | 1.22 | 1.285 | 0.269 | | | | |
|  | | pCO_2_ × Light | | | 0.139 | | 1,22 | 0.082 | 0.777 | | | | |
|  | |  | | |  | |  |  |  | | | | |
| PC2 (34.1%) | | pCO_2_ | | | 9.726 | | 1,22 | 5.502 | **0.028** | | | | |
|  |  | Light | | | 0.236 | | 1,22 | 0.133 | 0.718 | | | | |
|  | | pCO_2_ × Light | | | 0.019 | | 1,22 | 0.011 | 0.919 | | | | |
| Values in parentheses represent percentage of variation explained for each principal component (PC). *SS* = sum of squares; *df* = degrees of freedom in numerator and denominator; bold values represent significant effects (*p* < 0.05). | | | | | | | | | | | | | |

| **Table S3.** Statistical analysis of pCO_2_ and light effects on area-normalized net calcification, biomass energy reserves, *Symbiodinium* density, and photopigment concentrations of the reef coral *Pocillopora acuta*. | | | | | | | |
| --- | --- | --- | --- | --- | --- | --- | --- |
| *Dependent variable* | *Effect* | *SS* | *df* | | *F* | | *p* |
| calcification | pCO_2_ | 0.004 | | 1, 22 | 0.275 | 0.605 | |
| mg CaCO_3_ cm^-2^ d^-1^ | Light | 0.001 | | 1, 22 | 0.070 | 0.793 | |
|  | pCO_2_ × Light | 4.808 × 10^-4^ | | 1, 22 | 0.031 | 0.861 | |
|  |  |  | |  |  |  | |
| biomass mg cm^-2^ | pCO_2_ | 0.030 × 10^-4^ | | 1, 22 | 0.004 | 0.950 | |
|  | Light | 0.001 | | 1, 22 | 1.667 | 0.210 | |
|  | pCO_2_ × Light | 1.316 × 10^-4^ | | 1, 22 | 0.178 | 0.677 | |
|  |  |  | |  |  |  | |
| proteins mg cm^-2^ | pCO_2_ | 0.004 | | 1, 22 | 1.279 | 0.270 | |
|  | Light | 0.024 | | 1, 22 | 7.940 | **0.010** | |
|  | pCO_2_ × Light | 0.015 | | 1, 22 | 4.850 | **0.038** | |
|  |  |  | |  |  |  | |
| carbohydrates mg cm^-2^ | pCO_2_ | 0.002 | | 1, 22 | 0.909 | 0.351 | |
|  | Light | 0.001 | | 1, 22 | 0.459 | 0.505 | |
|  | pCO_2_ × Light | 0.005 | | 1, 22 | 2.453 | 0.132 | |
|  |  |  | |  |  |  | |
| lipids mg cm^-2^ | pCO_2_ | 0.402 | | 1, 22 | 2.283 | 0.145 | |
|  | Light | 0.018 | | 1, 22 | 0.104 | 0.751 | |
|  | pCO_2_ × Light | 0.030 | | 1, 22 | 0.171 | 0.683 | |
|  |  |  | |  |  |  | |
| energy content kJ cm^-2^ | pCO_2_ | 0.010 | | 1, 22 | 1.740 | 0.201 | |
|  | Light | 0.002 | | 1, 22 | 0.382 | 0.543 | |
|  | pCO_2_ × Light | 1.072 × 10^-4^ | | 1, 22 | 0.019 | 0.891 | |
|  |  |  | |  |  |  | |
| *Symbiodinium* cells cm^-2^ | pCO_2_ | 1.798 × 10^4^ | | 1, 22 | 0.962 | 0.338 | |
|  | Light | 4.791 × 10^4^ | | 1, 22 | 2.563 | 0.124 | |
|  | pCO_2_ × Light | 0.953 × 10^4^ | | 1, 22 | 0.510 | 0.483 | |
|  |  |  | |  |  |  | |
| chlorophyll *a* μg cm^-2^ | pCO_2_ | 1.000 × 10^-4^ | | 1, 21 | 1.000 × 10^-4^ | 0.993 | |
|  | Light | 25.085 | | 1, 21 | 31.055 | **<0.001** | |
|  | pCO_2_ × Light | 1.860 | | 1, 21 | 2.302 | 0.144 | |
|  |  |  | |  |  |  | |
| chlorophyll *c_2_* μg cm^-2^ | pCO_2_ | 0.130 × 10^-4^ | | 1, 22 | 0.002 | 0.961 | |
|  | Light | 0.104 | | 1, 22 | 18.894 | **<0.001** | |
|  | pCO_2_ × Light | 0.015 | | 1, 22 | 2.711 | 0.114 | |
|  |  |  | |  |  |  | |
| chlorophyll *a* pg cell^-1^ | pCO_2_ | 0.755 × 10^-4^ | | 1, 22 | 0.021 | 0.886 | |
|  | Light | 0.010 | | 1, 22 | 2.786 | 0.109 | |
|  | pCO_2_ × Light | 0.001 | | 1, 22 | 0.305 | 0.587 | |
|  |  |  | |  |  |  | |
| chlorophyll *c_2_* pg cell^-1^ | pCO_2_ | 4.362 × 10^-4^ | | 1, 22 | 0.092 | 0.765 | |
|  | Light | 0.008 | | 1, 22 | 1.618 | 0.217 | |
|  | pCO_2_ × Light | 0.003 | | 1, 22 | 0.594 | 0.449 | |
| *SS* = sum of squares; *df* = degrees of freedom in numerator and denominator; bold *P* values represent significant effects (*p* < 0.05) | | | | | | | |

| **Table S4.** Statistical analysis of pCO_2_ and light effects on biomass-normalized net calcification and biomass energy reserves of the reef coral *Pocillopora acuta*. | | | | | | |
| --- | --- | --- | --- | --- | --- | --- |
| *Dependent variable* | *Effect* | | *SS* | *df* | *F* | *p* |
| calcification | pCO_2_ | | 0.235 | 1, 22 | 0.306 | 0.586 |
| mg CaCO_3_ gdw^-1^ d^-1^ | Light | | 0.958 | 1, 22 | 1.243 | 0.277 |
|  | pCO_2_ × Light | | 0.018 | 1, 22 | 0.024 | 0.879 |
|  |  | |  |  |  |  |
| proteins g gdw^-1^ | pCO_2_ | | 0.680 × 10^-4^ | 1, 22 | 0.692 | 0.415 |
|  | Light | | 0.148 × 10^-4^ | 1, 22 | 0.150 | 0.702 |
|  | pCO_2_ × Light | | 0.480 × 10^-4^ | 1, 22 | 0.488 | 0.492 |
|  |  | |  |  |  |  |
| carbohydrates g gdw^-1^ | pCO_2_ | | 1.993 × 10^-4^ | 1, 22 | 0.943 | 0.342 |
|  | Light | | 0.001 | 1, 22 | 4.747 | **0.040** |
|  | pCO_2_ × Light | | 2.411 × 10^-4^ | 1, 22 | 1.141 | 0.297 |
|  |  | |  |  |  |  |
| lipids g gdw^-1^ | pCO_2_ | | 0.051 | 1, 22 | 4.762 | **0.040** |
|  | Light | | 0.007 | 1, 22 | 0.630 | 0.436 |
|  | pCO_2_ × Light | | 1.200 × 10^-4^ | 1, 22 | 0.011 | 0.917 |
|  |  | |  |  |  |  |
| energy content kJ gdw^-1^ | pCO_2_ | | 80.984 | 1, 22 | 4.721 | **0.041** |
|  | Light | | 10.385 | 1, 22 | 0.605 | 0.445 |
|  | pCO_2_ × Light | | 0.062 | 1, 22 | 0.004 | 0.952 |
|  |  |  | |  |  |  |
| *SS* = sum of squares; *df* = degrees of freedom in numerator and denominator; bold *P* values represent significant effects (*p* < 0.05). | | | | | | |

**3. Additional figure**

**Figure S1.** **Area-normalized (cm^-2^) (*a*) protein, (*b*) carbohydrates, (*c*) lipid biomass, and (*d*) tissue energy content in *Pocillopora acuta* corals exposed to light treatments (7.5 and 15.7 mol photons m^-2^ d^-1^) and ambient pCO_2_ (ACO_2_) and high pCO_2_ (HCO_2_)­ (*see* table 1).** Values displayed are means ± SE; *n* = 16 – 24 for lipid biomass and energy content, for other variables *n* = 28 (HL–HCO_2_) and *n* = 41 – 42 (all other treatments). *Asterisks* indicate a statistical difference (*p <* 0.05) between light treatments, and *individual letters* indicate no significant difference between treatments in post hoc multiple comparisons where pCO_2_ × light interactions were observed.

**References**

1. Schmidt-Roach, S., Miller, K. J., Lundgren, P. & Andreakis, N. 2014 With eyes wide open: a revision of species within and closely related to the Pocillopora damicornis species complex (Scleractinia; Pocilloporidae) using morphology and genetics. *Zool J Linn Soc-Lond* **170**, 1–33. (doi:10.1111/zoj.12092)

2. Cunning R, Ritson-Williams R, Gates RD. 2016 Patterns of bleaching and recovery of *Montipora capitata* in Kāne‘ohe Bay, Hawai‘i, USA. *Mar. Ecol. Prog. Ser.* **551**, 131–139. (doi:10.3354/meps11733)

3. Dickson AG, Sabine CL, Christian JR. 2007 *Guide to best practices for ocean CO_2_ measurements. PICES Special Publication 3; 2007*. http://cdiac.ornl.gov/oceans/Handbook_2007.html

4. Fitt WK, McFarland FK, Warner ME, Chilcoat GC. 2000 Seasonal patterns of tissue biomass and densities of symbiotic dinoflagellates in reef corals and relation to coral bleaching. *Limnol. Oceanogr.* **45**, 677–685. (doi:10.4319/lo.2000.45.3.0677)

5. Jeffrey SW, Humphrey GF. 1975 New spectrophotometric equations for determining chlorophylls a, b, c_1_ and c_2_ in higher plants, algae and natural phytoplankton. *Biochem. Physiol. Pflanz.* **167**, 191–194. (doi:10.1016/S0015-3796(17)30778-3)

6. Schoepf V *et al*. 2013 Coral energy reserves and calcification in a high-CO_2_ world at two temperatures. *PLoS ONE* **8**, e75049 (doi:10.1371/journal.pone.0075049.s003)

7. Dubois M, Gilles KA, Hamilton JK, Rebers P, Smith F. 1956 Colorimetric method for determination of sugars and related substances. *Anal. Chem.* **28**, 350–356. (doi:10.1021/ac60111a017)

8. Smith PK *et al*. 1985 Measurement of protein using bicinchoninic acid. *Anal. Bioch.* **150**, 76–85. (doi:10.1016/0003-2697(85)90442-7)

9. Gnaiger E, Bitterlich G. 1984 Proximate biochemical composition and caloric content calculated from elemental CHN analysis: a stoichiometric concept. *Oecologia* **62**, 289–298. (doi:10.1007/BF00384259)

10. Bates D, Mäechler M, Bolker BM, Walker SC. 2015 Fitting linear mixed-effects models using lme4. *J. Stat. Softw.* **67**, 1–48. (doi:10.18637/jss.v067.i01)

11. Kuznetsova A, Brockhoff PB, Christensen RHB. 2016 *lmerTest: Tests for random and fixed effects for linear mixed effect models.* R package version 2.0-33*.* https://cran.r-project.org/package=lmerTest

12. Lenth RV. 2016 Least-squares means: the R package lsmeans. *J. Stat. Softw.* **69**, 1–33. (doi:doi:10.18637/jss.v069.i01)(doi:doi:10.18637/jss.v069.i01)

13. Albright R, Mason B, Langdon C. 2008 Effect of aragonite saturation state on settlement and post-settlement growth of *Porites astreoides* larvae. *Coral Reefs* **27**, 485–490. (doi:10.1007/s00338-008-0392-5)

14. Drenkard EJ, Cohen AL, McCorkle DC, de Putron SJ, Starczak VR, Zicht AE. 2013 Calcification by juvenile corals under heterotrophy and elevated CO_2_. *Coral Reefs* **32**, 727–735. (doi:10.1007/s00338-013-1021-5)

15. Holcomb M, McCorkle DC, Cohen AL. 2010 Long-term effects of nutrient and CO_2_ enrichment on the temperate coral *Astrangia poculata* (Ellis and Solander, 1786). *J. Exp. Mar. Biol. Ecol.* **386**, 27–33. (doi:10.1016/j.jembe.2010.02.007)

16. Towle EK, Enochs IC, Langdon C. 2015 Threatened Caribbean coral is able to mitigate the adverse effects of ocean acidification on calcification by increasing feeding rate. *PLoS ONE* **10**, e0123394. (doi:10.1371/journal.pone.0123394.t003)

17. Krief S, Hendy EJ, Fine M, Yam R, Meibom A, Foster GL, Shemesh A. 2010 Physiological and isotopic responses of scleractinian corals to ocean acidification. *Geochim. Cosmochim. Ac.* **74**, 4988–5001. (doi:10.1016/j.gca.2010.05.023)

18. Reynaud S, Leclercq N, Romaine-Lioud S, Ferrier-Pagés C, Jaubert J, Gattuso J. 2003 Interacting effects of CO_2_ partial pressure and temperature on photosynthesis and calcification in a scleractinian coral. *Global Change Biol.* **9**, 1660–1668. (doi:10.1046/j.1365-2486.2003.00678.x)

19. Edmunds PJ, Brown D, Moriarty V. 2012 Interactive effects of ocean acidification and temperature on two scleractinian corals from Moorea, French Polynesia. *Global Change Biol.* **18**, 2173–2183. (doi:10.1111/j.1365-2486.2012.02695.x)

20. Dufault AM, Ninokawa A, Bramanti L, Cumbo VR, Fan TY, Edmunds PJ. 2013 The role of light in mediating the effects of ocean acidification on coral calcification. *J. Exp. Biol.* **216**, 1570–1577. (doi:10.1242/jeb.080549)

21. Vogel N, Meyer FW, Wild C, Uthicke S. 2015 Decreased light availability can amplify negative impacts of ocean acidification on calcifying coral reef organisms. *Mar. Ecol. Prog. Ser.* **521**, 49–61. (doi:10.3354/meps11088)

22. Marubini F, Barnett H, Langdon C, Atkinson MJ. 2001 Dependence of calcification on light and carbonate ion concentration for the hermatypic coral *Porites compressa*. *Mar. Ecol. Prog. Ser.* **220**, 153–162. (doi:10.3354/meps220153)

23. Suggett DJ, Dong LF, Lawson T, Lawrenz E, Torres L, Smith DJ. 2013 Light availability determines susceptibility of reef building corals to ocean acidification. *Coral Reefs* **32**, 327–337. (doi:10.1007/s00338-012-0996-7)

24. Comeau S, Carpenter RC, Edmunds PJ. 2014 Effects of irradiance on the response of the coral *Acropora pulchra* and the calcifying alga *Hydrolithon reinboldii* to temperature elevation and ocean acidification. *J. Exp. Mar. Biol. Ecol.* **453**, 28–35. (doi:10.1016/j.jembe.2013.12.013)

25. Comeau S, Carpenter RC, Edmunds PJ. 2013 Effects of feeding and light intensity on the response of the coral *Porites rus* to ocean acidification. *Mar. Biol.* **160**, 1127–1134. (doi:10.1007/s00227-012-2165-5)
